# Berberine, a natural compound, suppresses Hedgehog signaling pathway activity and cancer growth

**DOI:** 10.1186/s12885-015-1596-z

**Published:** 2015-08-22

**Authors:** Juan Wang, Yuanqiu Peng, Yuan Liu, Jun Yang, Ning Ding, Wenfu Tan

**Affiliations:** 1 Department of Pharmacology, School of Pharmacy, Fudan University, 826 Zhangheng Road, Shanghai, 201203 P.R. China; 2 Department of Medicinal Chemistry, School of Pharmacy, Fudan University, 826 Zhangheng Rd, Shanghai, 201203 P.R. China; 3 State key Laboratory of Drug Research, Shanghai Institute of Materia Medica, Chinese Academy of Sciences, Shanghai, 201203 China

## Abstract

**Background:**

Berberine (BBR), a natural alkaloid compound, is used as a non-prescription drug in China for treating diarrhea and gastroenteritis. Many studies have revealed that BBR possesses anticancer effect. However, the molecular mechanisms underlying its anticancer action is far from being fully elucidated. This study is aimed to determine the effect of BBR on the hedgehog (Hh) activity and the growth of cancers addiction to Hh activity.

**Methods:**

The Hh activity was determined by dual luciferase assays and quantitative RT-PCR analyses. The growth inhibition of BBR on medulloblastoma which was obtained from ptch+/−;p53−/− mice was analyzed by 5-bromo-2-deoxyuridine (Brdu) assays and by allografting the medulloblastoma into nude mice. The data were statistically analyzed by one-way analysis of variance (ANOVA), and multiple comparison between the groups was performed using Dunnett’s method.

**Results:**

In this study, we found that BBR significantly inhibited the Hh pathway activity. Meanwhile, we observed that BBR failed to affect the transcriptional factors activities provoked by tumor necrosis factor-α (TNF-α) and Prostaglandin E2 (PGE2), thus suggesting its unique property against Hh pathway activity. Further studies revealed that BBR inhibited the Hh pathway activity by potentially targeting the critical component Smoothened (Smo) and most likely shared the same binding site on Smo with cyclopamine, a classical Smo inhibitor. Finally, we demonstrated that BBR obviously suppressed the Hh-dependent medulloblastoma growth *in vitro* and *in vivo*.

**Conclusion:**

Collectively, our study uncovered a novel molecular mechanism responsible for the anticancer action of BBR, thus opening the way for the usage of BBR for therapeutics of cancers addiction to aberrant Hh pathway activity.

## Background

Hh signaling pathway is an evolutionarily conserved signaling axis of embryonic patterning and tissue homeostasis [1, 2]. Deregulated activity of the Hh signaling pathway has also been shown to be involved in the development of tumors which arise sporadically or in genetically predisposed individuals. In vertebrates, three Hh ligands (Sonic hedgehog, Shh; Indian hedgehog, Ihh; Desert hedgehog, Dhh) have been identified that bind to the 12-transmembrane cell surface receptor Patched1 (PTCH1). When not bound by Hh ligands, PTCH1 restrains the activity of Smo, a member of the 7-transmemebrane cell surface receptor. However, upon bound by a ligand, PTCH1 no longer inhibits Smo, thus allowing the accumulation of Smo in the primary cilium. Ultimately, canonical Hh signaling regulates the activity, proteolytic processing, and the stability of the Gli family transcriptional factors, Gli1-3, and subsequently initiates the transcription of Gli-dependent target genes, such as *Gli1* and *ptch1* [3]. This regulation requires a number of protein kinases, including protein kinase A, glycogen synthase kinase 3 and casein kinase 1, and the negative regulator suppressor of fused (SuFu) [4].

The mechanisms responsible for the constitutive Hh pathway activity in cancers include ligand-independent and ligand-dependent manner. Ligand-independent constitutive activation of Hh pathway in cancers is characterized by somatic mutations in *Ptch1*, *Smo*, or *Sufu*, consequently increasing Hh pathway activity and leading to a higher incidence of basal cellular carcinoma (BCC) and medulloblastoma. On the other hand, aberrant Hh pathway activity in a wide range of cancers is associated with elevated Hh ligands secretion from tumors or the stromal environment, namely ligand-dependent manner [2, 5]. Large-scale efforts have been made to develop Hh pathway antagonists for treatment of cancers. As a central regulator of the pathway and a readily accessible cell surface component, Smo has been the most successful target for developing Hh antagonists. There are currently nine novel Smo inhibitors in clinic trial [6, 7]. Among them, vismodegib has been approved for treating in advanced unresectable BCC in 2012 [8].

**Fig. 1:**
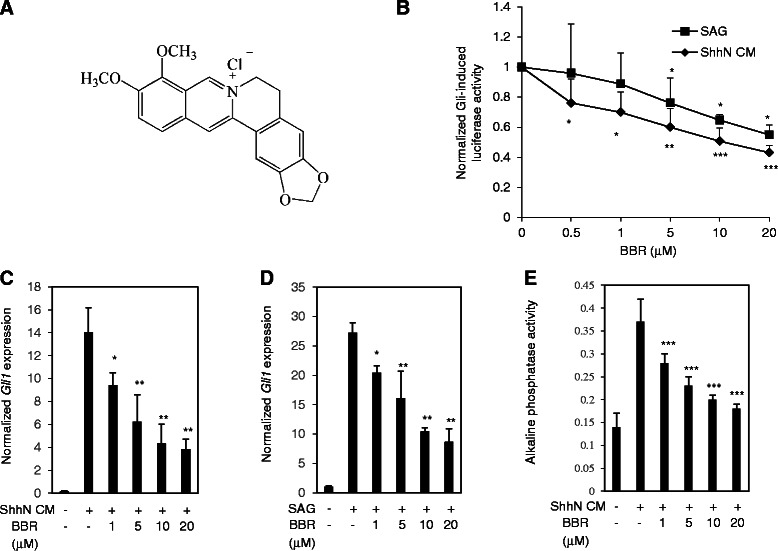
BBR inhibits Hh pathway activity *in vitro*. **a** Chemical structure of BBR. **b** BBR inhibited the Gli luciferase activity provoked by ShhN CM and SAG. NIH-3 T3 cells transfected with luciferase reporter plasmids containing 8 × Gli binding sites and Renilla-TK plasmids were exposed to ShhN CM or SAG supplemented with various concentrations of BBR for 36 h. The cells were lysed for the dual luciferase reporter activity analysis. The firefly luciferase activity was normalized by the *Renilla* luciferase activity. The results are expressed as means ± s.d. from three independent experiments (*n* = 3). (**c**-**d**) BBR inhibited the mRNA expressions of Gli1 provoked by ShhN CM (**c**) and SAG (**d**). NIH-3 T3 cells were treated with ShhN CM or SAG with or without various concentrations of BBR for 24 h and harvested for RT-qPCR analysis. Data are expressed as mean ± s.d. (*n* = 3). Experiments were repeated for three times. **e** BBR inhibited alkaline phosphatase activity in C3H10T1/2 cells. C3H10T1/2 cells were treated with or without ShhN CM supplemented with various concentrations of BBR for 72 h. The results are expressed as mean ± s.d. from three independent experiments (*n* = 3)

BBR (chemical structure shown in Fig. 1a), a natural isoquinoline alkaloid, can be isolated from the rhizome, roots and stem barks of various important medicinal plants, the *Berberis* species. BBR exhibits multiple pharmacological activities, such as antimicrobial, antidiabetic, cardioprotective effects [9]. Additionally, it has been shown that BBR may inhibit the growth of a variety of human cancer cell lines, including prostate [4, 10], colon cancer [11], lung cancer [12, 13], nasopharyngeal cancer [14], breast cancer [15, 16], and leukemia cells [17]. However, the molecular mechanisms underlying the anticancer effect of BBR remain far from being fully elucidated. In this study, we identified that BBR may selectively inhibit the Hh signaling pathway activity by targeting Smo and consequently the Hh-dependent cancer growth, thus improving our knowledge of the molecular mechanisms responsible for the anticancer action of BBR and contributing to the future usage of BBR as an anticancer drugs.

## Methods

### Cell lines and culture

The NIH-3T3 and C3H/10T1/2 mouse embryo fibroblast cells, HEK-293T human epithelial kidney cells, and LS174T colon cancer cells were obtained from the American Type Culture Collection (Manassas, VA). All these cells were routinely cultured according to the manufacturer’s instructions.

The variant containing the N-terminal signaling domain of the Shh (Shh) conditioned medium (CM) were prepared as previously described [18]. Briefly, 293 T cells (5 × 10^6^) were seeded in 10-cm dishes. The plasmid harboring the ShhN were transfected into 293 T cells with Lipofectamine 2000 reagent (Invitrogene; Grand Island, NY). The medium (5 ml) in the cells were replaced with fresh medium with 0.1 % serum 24 h post transfection. After 24, the ShhN CM were collected, and were diluted 100-fold prior to be used for experiments.

### Reagents and antibodies

BBR and PGE2 were obtained from Sigma-Aldrich (St. Louis, MO). The Hh pathway antagonists cyclopamine, GANT61, itraconazole, and BODIPY-cyclopamine were obtained from Biovision (Milpitas, CA). The Hh pathway agonist SAG was obtained from Selleck Chemicals (Houston, TX). TNF-α, BAY 11–8072 and H89 were purchased from Beyotime (Suzhou, China). Primary antibodies against Smo, Gli2, and Sufu and GAPDH were obtained from Santa Cruz Biotechnology (Santa Cruz, CA).

### Plasmids and lentivirus

The 8 × Gli1-binding site luciferase reporter (8 × GBS-luciferase) plasmid was a kind gift from Dr. Hiroshi Sasaki. The TCF/LEF-luciferase reporter plasmid, NF-κB –luciferase reporter plasmid, and TK-Renilla luciferase plasmid were purchased from Promega (Madison, WI). The Gli2 lacking the N-termianl (Gli2ΔN) plasmid and ShhN plasmids were obtained from Addgene (Cambridge, MA). The Myc-DKK-tagged ORF clone of Homo sapiens Smo plasmid was purchased from Origene (Rockville, MD). The mutant human plasmid SmoM2 (W535L) was generated from wild type Smo plasmids using QuickChange Site-Directed Mutagenesis kit from Agilent (Santa Clara, CA) and was confirmed by sequencing. The Sufu-shRNA was purchased from Santa Cruz (Santa Cruz, CA).

Transient transfections were performed using Lipofectamine 2000 reagent from Invitrogen according to the manufacturer’s instructions. The lentiviral stocks were prepared according to previous report [19]. Briefly, the plasmid carrying the Sufu-shRNA and three packaging plasmids were co-transfected into 293T cells using Lipofectamine 2000. The viruses were harvested 24 h post transfection and 4 ml viruses were used for infected NIH-3T3 cells seeded in 10-cm dishes. Infected cells were analyzed 5–7 days post infection by western blot analyses of the expression of Sufu.

### Dual luciferase assays

Cells transfected with luciferase reporter plasmids containing respective binding-sites of various transcriptional factors and Renilla-TK construct were seeded into 48-well plates. After various treatments as indicated, luciferase assays were conducted using a dual luciferase assay kit according to the manufacturer’s instructions (Promega) on a luminometer (Molecular Device; Sunnyvale, CA). The firefly luciferase values were normalized to *Renilla* values.

### Reverse transcription and quantitative polymerase chain reaction (RT-qPCR)

Total RNA was extracted from cells or medullbolbatoma tissues using Trizol reagent (Takara; Dalian, China) following the manufacturer’s protocol. The qPCR analyses were performed using the following primers:

- mGUSB: Forward: 5′-CTGCCACGGCGATGGA-3′
  - Reverse: 5′-ACTGCATAATAATGGGCACTGTTG-3′
- mGli1: Forward: 5′-GCAGTGGGTAACATGAGTGTCT-3′
  - Reverse: 5′-AGGCACTAGAGTTGAGGAATTGT-3′
- mptch1: Forward: 5′–GCTACGACTATGTCTCTCACATCAACT-3′
  - Reverse: 5′-GGCGACACTTTGATGAACCA-3′

The mRNA levels of interested genes were normalized to those of GUSB.

### Western blot analysis

NIH-3T3 cells were harvested for western blot analysis of the expression of Smo, Gli2, and Sufu according to standard procedure. The blots of GAPDH were used as loading controls.

### Alkaline phosphatase activity assay

C3H10T1/2 cells were plated into 96-well plates at a density of 5000 cells per well. After treatment with or without ShhN CM supplemented with various concentrations of BBR for 72 h. The alkaline phosphatase activity was measured using a kit from Beyotime on a plate reader (Molecular Device) at 405 nm.

### Fluorescent BODIPY-cyclopamine competition assay

The 293T cells were seeded onto coverslips coated with poly-D-lysine in 24-well plates, followed by transfection with hSMO construct. After exposed to 1 uM BODIPY-cyclopamine supplemented with or without various compounds as indicated for 10 h, the cells were washed with PBS, fixed with paraformaldehyde (4 %; *v*/*v*) for 10 min, incubated with a 0.1 % Triton X-100 for 15 min. The cells were then subjected to fluorescence-activated cell sorting (FACS; Becton Dickinson; San Jose, CA) analysis or were mounted with DAPI and visualized using a fluorescence microscope (Leica; Wetzlar, Germany).

### Medulloblastoma cells culture and Brdu assay

Medulloblastoma cells were obtained from medulloblastoma allografts by mechanical dissociation and viable cell fractioning. The cells were maintained in Neurobasal A medium (Invitrogen) containing B-27 supplement (Invitrogen), epidermal growth factor 20 ng/ml (Invitrogen), basic fibroblastic growth factor 20 ng/ml (Invitrogen), nonessential amino acids (Invitrogen), N-acetyl cysteine 60 mg/ml, and Glutamax (Invitrogen) [20].

Medulloblastoma cells were seeded into 96-well plates, followed by treatment with various concentrations of BBR for 36 h. The Brdu assays were conducted using the Brdu Cell Proliferation Kit (Merck Millipore; Bedford, MA) according to manufacturer’s instructions.

### Medulloblastoma allograft model

Primary intracranial medulloblastoma from Ptch+/−; p53−/− mouse [21, 22], which was obtained by crossing ptch+/− mice (Jackson Laboratory; Harbor, MI) with p53−/− mice (Jackson Laboratory), were harvested and allografted subcutaneously into right and left flanks of athymic nude mice (Beijing HFK Bio-Technology; Beijing, China). The well-developed tumors were harvested, cut into 1 mm^3^ fragments and inoculated subcutaneously into the right flank of athymic nude mice using a trocar. When the tumor volume reached 100–150 mm^3^, the mice were randomly assigned into control and treatment group (*n* = 8). Control group was given vehicle alone, and treatment group received BBR (100 mg/kg) via oral gavage once daily for 3 weeks. The volume of the tumors were measured twice per week using microcaliper. The tumor volume (V) was calculated as following: V = [length(mm) × width^2^(mm^2^)]/2. The individual relative tumor volume (RTV) was calculated as following: RTV = Vt/V0, where Vt is the volume on each day, and V0 represents the volume at the beginning of the treatment. All animal experiments in this study were pre-approved by the Animal Care and Use Committee of the Fudan University and performed according to institutional policies.

### Statistical analysis

Statistical differences were analyzed by one-way ANOVA and *P* < 0.05 was considered as significant. Multiple comparison between the groups was performed using Dunnett’s method. (#*p* > 0.05; *P < 0.05, **P < 0.01, *** < 0.001).

## Results

### BBR inhibits Hh signaling *in vitro*

To determine whether BBR can inhibit the Hh signaling, NIH-3T3 cells, which have good response to the Hh stimulation [23], transfected with 8 × Gli1-binding site dependent firefly luciferase and Renilla Luciferase plasmids were stimulated with ShhN CM with or without various concentrations of BBR. We observed that BBR obviously inhibited the Gli-responsive reporter activity provoked by ShhN CM in a dose-dependent manner (Fig. 1b), with an IC50 value of 4.6 ± 1.2 μM. Concomitantly, BBR reduced the mRNA expression of *Gli1* (Fig. 1c), a transcriptional target of Gli, which served as a readout of Gli activity. Moreover, we found that BBR treatment also abolished the Gli luciferase activity (Fig. 1b) and Gli1 mRNA abundance (Fig. 1d) provoked by SAG, a small molecular compound agonist of Smo [24]. To further determine the ability of BBR of suppressing the Hh pathway activity, we conducted the alkaline phosphatase activity assay using C3H10T1/2 cells, which can express osteogenesis marker alkaline phosphatase when treated with Hh ligands [25, 26]. As shown in Fig. 1e, exposure of BBR obviously suppressed the alkaline phosphatase activity evoked by ShhN CM in C3H10T1/2 cells. The inhibitory effect of BBR on the alkaline phosphatase activity was not due to the non-specific cytotoxic activity of BBR, as BBR had no effect on the cell numbers of C3H10T1/2 cells after BBR treatment for 72 h (data not shown). Hence, our data show that BBR may significantly inhibit the Hh signaling *in vitro*.

### BBR displays selectivity for inhibiting Hh pathway activity

**Fig. 2:**
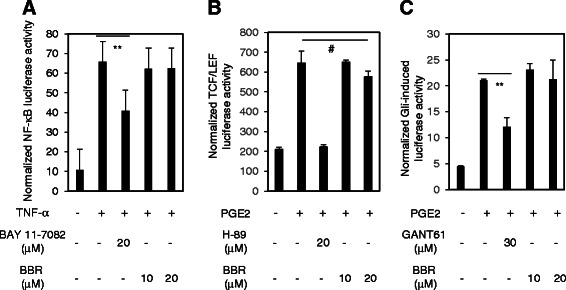
BBR displays selectivity for inhibiting Hh pathway activity. **a** The 293 T cells transfected with luciferase reporter activity containing NF-κB binding sites and Renilla-TK plasmids were exposed to TNF-α with BAY 11-872 or various concentrations of BBR for 6 h, and the cells were harvested for dual luciferase reporter activity analysis. The data are expressed as mean ± s.d., (*n* = 3) from three independent experiments. **b** The LS174T cells transfected with luciferase reporter constructs containing TCF/LEF binding sites and the Renilla-TK plasmids were exposed to PGE2 with various concentrations of BBR or H89 for 24 h, and the cells were collected for dual luciferase reporter activity analysis. The results are expressed as means ± s.d. from three independent experiments (*n* = 3). **c** The LS174T cells transfected with luciferase reporter constructs containing Gli binding sites and the Renilla-TK plasmids were exposed to PGE2 with various concentrations of BBR or GANT61 for 24 h, and the cells were collected for dual luciferase reporter activity analysis. Data are expressed as means ± s.d. from three independent experiments (*n* = 3). *p < 0.05; #*p* > 0.05

To rule out the possibility that BBR nonspecifically inhibits Gli luciferase activity provoked by ShhN CM, we examined the effect of BBR on other transcriptional factors, such as NF-κB, and TCF/LEF [27]. As shown in Fig. 2a-b, we observed that tumor necrosis factor α (TNF-α) and Prostaglandin E2 (PGE2) obviously provoked the NF-κB, and TCF/LEF luciferase activity, while BBR exhibited no inhibitory activity against either NF-κB, or TCF/LEF luciferase activity stimulated by TNF-α and PGE2. The BAY 11-7082, and H89 were used as positive controls for inhibition of NF-κB, and TCF/LEF luciferase activity, respectively (Fig. 2a-b). We have demonstrated that PGE2 may activate the Gli activity in a noncanonical manner (data to be published). We then asked whether BBR may suppress the Gli activity provoked by PGE2. Interestingly, BBR failed to inhibit the Gli luciferase activity activated by PGE2 (Fig. 2c). However, the GANT61, a small molecule inhibitor targeting Gli [28], abundantly reduced the Gli luciferase activity stimulated by PGE2 (Fig. 2c). Hence, our data demonstrate that BBR exhibits unique property for suppressing Hh signaling pathway activity.

### BBR inhibits the Hh signaling pathway activity by targeting Smo

**Fig. 3:**
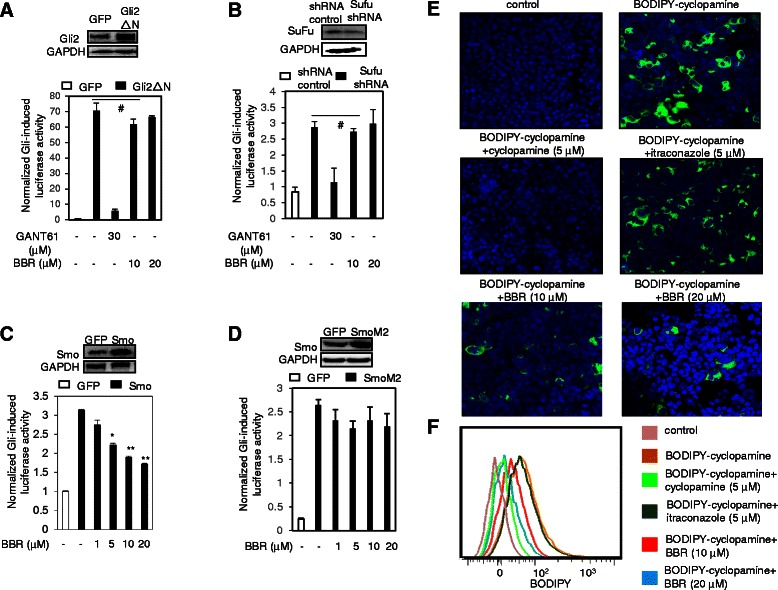
BBR inhibits the Hh signaling pathway activity by potentially targeting Smo. a BBR had no effect on the Gli luciferase activity stimulated by ectopic expression of Gli2△N. NIH-3 T3 cells transfected with Gli2△N, Gli luciferase reporter and the Renilla-TK constructs were treated with GANT61 and various concentrations of BBR for 36 h. GFP was used as a control for Gli2△N. The results are expressed as mean ± s.d. from three independent experiments (*n* = 3). **b** BBR exhibited no effect on the Gli luciferase activity provoked by knockdown of Sufu. NIH-3 T3 cells were infected with lenti-virus harboring Sufu shRNA or shRNA control (the inserts shows the efficacy of knockdown by western blot analysis). The cells were further transfected with Gli luciferase reporter and the Renilla-TK constructs, and exposed to GANT61 or BBR for 36 h. Data are expressed as means ± s.d. (*n* = 3). (**c**-**d**) BBR significantly inhibited the Gli luciferase activity provoked by ectopic expression of Smo (**c**), while exhibited no effect on that provoked by ectopic expression of SmoM2 (**d**). NIH-3 T3 cells were transfected with Smo or SmoM2, Gli luciferase reporter and the Renilla-TK constructs were treated with various concentrations of BBR. GFP were used as a control for Smo or SmoM2. The results are expressed as mean ± s.d. from three independent experiments (*n* = 3). **e** BODIPY-cyclopamine competition analysis. Photographs are representatives from three distinct experiments. 293 T cells transfected with hSMO expression construct were exposed to 1 μM BODIPY-cyclopamine supplemented with or without cyclopamine, itraconazole or BBR for 10 h. The cells were then mounted with DAPI and photographed. Photographs are representatives from three distinct experiments (**f**). BODIPY-cyclopamine bound to Smo in 293 T cells as monitored by FACS analysis. #*p* > 0.05

Having demonstrated that BBR may specifically inhibit the Hh signaling pathway activity, we then set out to determine the molecular target of BBR for inhibiting Hh pathway activity. We first tested whether BBR may suppress the Gli luciferase activity provoked by ectopic expression of Gli. As shown in Fig. 3a, overexpression of Gli2ΔN (Fig. 3a, upper panel), a Gli2 variant missing 328 N-terminal amino acids, in the NIH-3T3 cells obviously stimulated the Gli-luciferase activity. However, BBR had no inhibitory effect on the Gli luciferase activity provoked by Gli2ΔN, while the Gli small molecular compound inhibitor GANT61 (Fig. 3a), which was used as a positive control, significantly suppressed the Gli luciferase activity. Thus, these observations ruled out the possibility that BBR inhibited the Hh signaling pathway activity by targeting Gli. Moreover, BBR also failed to affect the Gli-luciferase activity provoked by limiting the expression of Sufu (Fig. 3b), a negative regulator of Hh signaling pathway [28], suggesting that BBR inhibited the Hh pathway activity by acting upstream of Sufu. Based on these findings, we asked whether BBR inhibited the Hh pathway activity by targeting Smo, which is the most successful molecular target for developing anti-cancer drugs in Hh pathway. To this end, we examined the effect of BBR on the Gli-luciferase activity stimulated by ectopic expression of Smo in NIH-3T3 cells N (Fig. 3c, upper panel). BBR obviously inhibited the Gli-luciferase activity stimulated by ectopic expression of Smo (Fig. 3c), with the IC50 value (5.1 ± 3.4 μM) similar to that for inhibiting the Gli-luciferase activity stimulated by ShhN CM (4.6 ± 1.2 μM). However, BBR exhibited little effect on the Gli-luciferase activity stimulated by SmoM2 (Fig. 3d), a frequent mutant found in cancers [29]. These data suggest that BBR inhibits the Hh pathway activity potentially through targeting Smo.

Furthermore, as shown in Fig. 1b, d, we saw that BBR also significantly inhibited the Gli-luciferase activity (Fig. 1b) and Gli1 mRNA expression (Fig. 1d) provoked by SAG, a small molecular compound agonist of Smo [24]. Interestingly, the IC50 value of BBR for inhibiting the Gli-luciferase activity provoked by ShhN CM (4.6 ± 1.2 μM.) is lower than that for inhibiting Gli-luciferase activity provoked by SAG (8.7 ± 1.2 μM). Given that SAG and cyclopamine bind to the same site on Smo [24], these data suggest that BBR functions as a competitive inhibitor with SAG and most likely share the same binding site on Smo with cyclopamine. To further strengthen this argument, we used the BODIPY-cyclopamine, a fluorescent cyclopamine derivative, assay. We observed that the binding of BODIPY-cyclopamine to Smo was significantly suppressed by BBR, similar to cyclopamine, which herein served as a positive control (Fig. 3e). However, itraconazole, a Smo inhibitor binding to Smo on a distinct binding site from that of cyclopamine [20], failed to affect the binding of BODIPY-cyclopamine to Smo (Fig. 3e). These observations were further confirmed by FACS analysis (Fig. 3f). Taken together, our data demonstrate that BBR acts on Smo to inhibit the Hh pathway.

### BBR inhibits the Hh-dependent medulloblastoma cell growth *in vitro*

**Fig. 4:**
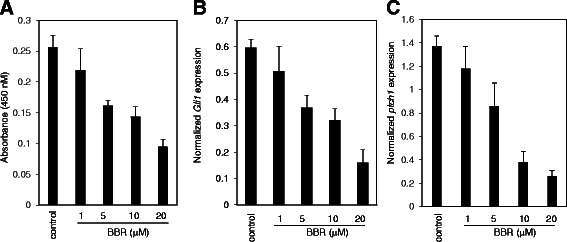
BBR suppresses the proliferation of medulloblastoma cells *in vitro*. **a** BBR suppressed the proliferation of medulloblastoma cells analyzed by Brdu assay. Medulloblastoma cells were treated with various concentrations of BBR for 36 h. Data are expressed as means ± s.d. from three independent experiments (*n* = 3). (**b**-**c**) BBR suppressed the *Gli1* mRNA (**b**) and *ptch1* mRNA (**c**) expression in medulloblastoma cells. Medulloblastoma cells were exposed to various concentrations of BBR for 24 h, and the cells were collected for RT-qPCR analysis. The results are expressed as mean ± s.d. from three independent experiments (*n* = 3)

Having characterized the Hh pathway inhibitory activity of BBR, we examined the effect of BBR on the growth of Hh-dependent medulloblastoma cells isolated from medulloblastoma in ptch+/−;p53−/− mice [21, 22]. Brdu assay revealed that BBR dose-dependently inhibited the growth of medulloblastoma cell growth (Fig. 4a), concomitantly with comparable reduction of the mRNA expression of Gli1, and ptch1 (Fig. 4b, c), which served as a readout of the Hh pathway activity. Hence, our data show that BBR may inhibit the Hh-dependent medulloblastoma cells growth *in vitro* through inhibiting the Hh pathway activity.

### BBR inhibits the Hh-dependent medulloblastoma growth *in vivo*

**Fig. 5:**
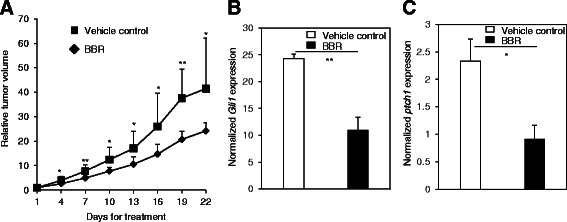
BBR inhibits the growth of medullboblastoma *in vivo*. **a** Inhibitory effect of BBR on the growth of medulloblastoma *in vivo*. Nude mice allografted with medulloblastoma were administered the BBR 100 mg/kg by daily gavage for 21 days. RTV for indicated days was shown as mean ± s.d. for each group of mice. (**b**-**c**) BBR inhibited the Gli1 mRNA (**b**) and *ptch1* mRNA (**c**) expression in the medulloblastoma tissues isolated from nude mice. Four hours after last dose of BBR administration, the medulloblastom tissues were harvested for RT-qPCR analysis. Data are expressed as mean ± s.d. *p < 0.05

To further demonstrate that BBR may inhibit the growth of the Hh-dependent medulloblastoma growth, we allografted the medulloblastoma isolated from ptch+/−;p53−/− mice into the Nude mice. BBR was administrated by gavage by 100 mg/kg daily. Consistent with the *in vitr*o data, BBR significantly inhibited the medulloblastoma growth (Fig. 5a), which is accompanied with similar reduction of the mRNA expression of Gli1 (Fig. 5b), and ptch1 (Fig. 5c). Hence, our *in vivo* data further demonstrate that BBR may inhibit the growth of Hh-dependent medulloblastoma growth by inhibiting the Hh pathway activity.

## Discussion

BBR has strong anti-inflammatory and antimicrobial activities. Owing to its excellent safety characteristics, BBR has been used as a non-prescription drug to treat diarrhea and gastroenteritis in China since 1950s. Accumulating reports show that BBR also possesses anti-cancer effects, being able to suppress the proliferation of cancer cells [9]. Previous studies have revealed various potential molecular targets responsible for the anticancer actions of BBR, such as DNA topoisomerase [30], HIF-1α [31], wnt signaling pathway [11]. In this study, we demonstrate that BBR may selectively suppress the Hh signaling pathway activity by potentially targeting the critical component Smo, and consequently inhibit the Hh-dependent cancer growth. Therefore, our study improve our knowledge about the underlying molecular mechanisms behind the anticancer actions of BBR, thus contributing to the future usage of BBR for treating cancers driven by aberrant Hh pathway activity.

This study shows that BBR displays specificity when inhibiting Hh pathway activity, as reflected by no inhibitory effects of BBR on the NF-κB, TCF/LEF and Gli acitivity in response to TNF-α and PGE2. However, other labs have shown that BBR may suppress the NF-kB activity which consequently induces the apoptosis of multiple myeloma cells or protects against neuronal damage via suppression of glia-mediated inflammation in traumatic brain injury [32, 33]. This discrepancy between our observation and those from other labs [32, 33] may be due to the distinct concentrations of BBR used. The maximum concentration used in the present study was 20 μM, while the concentrations which had inhibitory effect on the NF-κB activity in the studies from other labs ranged from 50 to 100 μM [32, 33].

Addiction of tumors to Hh signaling pathway for growth and metastasis has been verified with various Hh pathway inhibitors. The natural teratogenic compound cyclopamine, the first identified Hh pathway inhibitor, blocks Hh pathway by directly binding to Smo, slowing down tumor growth in animal models [34]. However, concerns were raised on its anticancer efficacy due to the off-target potential [35]. Moreover, the limited potency and poor oral solubility hinders its clinical development. Other more potent and selective Smo inhibitors with diverse chemical structures have been developed and are being investigated in clinical trials in a large range of advanced and metastatic cancers, such as vismodegib, sonidegib, BMS-833923, PF04449913 and LY2940680 [7]. Vismodegib has been approved for treatment of advanced BCC in 2012 [8], therefore underpinning the Smo as a molecular target for treating cancers. Among all these Smo inhibitors, the majority share the same binding site in Smo with cyclopamine, with the exceptions of itraconazole and several other antagonists newly discovered by Tao et al. [36]. In the present study, we identified that BBR can inhibit the Hh pathway activity by targeting Smo. We further identified that BBR may most likely bind Smo at the same pocket with cyclopamine, as reflected by competition of BBR for binding to Smo with SAG and BODIPY-cyclopamine. Of course, we cannot exclude the possibility that Smo acts on Smo indirectly via another molecule, as lacking direct binding assay. Hence, further verification of this argument by direct binding assay will help us to understand the characteristics of the BBR action on Smo.

## Conclusion

In summary, this study shows that BBR may selectively inhibit the Hh pathway activity functioning as a Smo inhibitor potentially most likely by binding the same site in Smo with cyclopamine. Using the ptch+/−;p53−/− medulloblastoma model, we also demonstrate that BBR significantly inhibit the Hh-dependent tumor growth by inhibiting Hh pathway activity. Given that BBR has been used as a non-prescription drug in China for decades, and it has a good safety profile, it should be possible for us to quickly investigate its clinical efficacy on patients with tumors dependent on the Hh pathway by either alone or in combination with other treatment strategies.

## Abbreviations

ANOVA: Analysis of variance
BBR: Berberine
BCC: Basal cellular carcinoma
Brdu: 5-bromo-2-deoxyuridine
CM: Conditioned medium
Dhh: Desert hedgehog
FACS: Fluorescence-activated cell sorting
Gli2ΔN: Gli2 lacking the N-termianl
Hh: Hedgehog
Ihh: Indian hedgehog
PGE2: Prostaglandin E2
PTCH1: Patched1
RT-qPCR: Reverse transcription and quantitative polymerase chain reaction
RTV: Relative tumor volume
Shh: Sonic hedgehog
ShhN: The variant containing the N-terminal signaling domain of the Shh
Smo: Smoothened
SuFu: Suppressor of fused
TNF-α: Tumor necrosis factor-α
